# Potential impacts of the ring nematode, *Mesocriconema xenoplax*, on grapevines in British Columbia: a microplot study

**DOI:** 10.21307/jofnem-2020-086

**Published:** 2020-08-18

**Authors:** Thomas Forge, Rosanne Smit, Denise Neilsen, Gerry Neilsen

**Affiliations:** 1 Agriculture and Agri-Food Canada, 4200 Hwy 97, Summerland, BC V0H 1Z0, Canada; 2 Carnation Farms, 28901 NE Carnation Farms Road, Carnation, WA, 98014

**Keywords:** Grape rootstock, *Mesocriconema xenoplax*, Nematode ecology, Nematode management, *Vitis*

## Abstract

The Okanagan Valley of British Columbia hosts a wine grape industry that has grown substantially in the past three decades in terms of both acreage and economic benefit to the region. The ring nematode, *Mesocriconema xenoplax*, has recently been found to be widespread in vineyard soils in the region. This study used field microplots to assess the potential impacts of a local population of *M. xenoplax* on the first four years growth of either self-rooted ‘Merlot’ or ‘Merlot’ vines grafted onto three commonly used rootstocks: 3309C, 44-53M, and Riparia Gloire. The population of *M. xenoplax* multiplied to comparable levels on self-rooted vines and all rootstocks, indicating that none of the vine genotypes were resistant to *M. xenoplax*. Inoculation with *M. xenoplax* reduced cumulative pruning weights of self-rooted vines by 58%. Inoculation with *M. xenoplax* reduced trunk cross-sectional areas of 3309C by 45% and that of self-rooted vines by 38%, whereas it did not affect trunk cross-sectional areas of 44-53 or Riparia Gloire, indicating differing levels of rootstock tolerance to *M. xenoplax*. Our data suggest that *M. xenoplax* is likely impacting vineyard health and productivity in the region, and the selection of rootstocks and management practices to minimize impacts of this nematode should be considered in future vineyard replant management programs.

The wine grape industry in the Okanagan Valley of British Columbia (BC), Canada has grown rapidly with respect to acreage and economic value since the early 1990s, with approximately 4,000 ha of *Vitis vinifera* varieties now producing an estimated $2.8 billion total return to the BC economy when agri-tourism benefits are included (https://news.gov.bc.ca/factsheets/factsheet-british-columbias-wine-industry). Since approximately 2005 when local diagnostic labs adopted sugar-flotation nematode extraction techniques, the ring nematode, *Mesocriconema xenoplax,* has been found to be increasingly prevalent in vineyard soils in the region, and a recent systematic survey (Forge et al., 2019) indicated that *M. xenoplax* is present in nearly 80% of BC vineyards.

Previous research utilizing field microplots in Oregon (Pinkerton et al., 2004, 2005; Schreiner, Pinkerton and Zasada, 2012; Schreiner, Zasada and Pinkerton 2012) and California (McKenry et al., 2001) demonstrated that *M. xenoplax* can have significant impacts on early growth of self-rooted cultivars of *V. vinifera* and a wide range of grape rootstocks. A greenhouse study (Pinkerton et al., 2005) indicated that most commercial rootstocks were hosts for an Oregon population of *M. xenoplax,* but that they tended to be more resistant and tolerant than self-rooted vines; only 420A and 101-14 rootstocks were classified as resistant and moderately resistant, respectively. Both 420A and 101-14 were susceptible, however, to a California population of *M. xenoplax* (Pinkerton et al., 2005), indicating the possibility of differences in aggressiveness between geographically distinct populations of *M. xenoplax*.

Given the differences in growing conditions between BC and coastal Oregon and California, and the potential for geographical differences in *M. xenoplax* populations, it is not clear if *M. xenoplax* poses a significant threat to the wine grape industry in BC. The objective of this study was to use a field microplot approach to measure the impacts of a BC population of *M. xenoplax* on the first four years growth of self-rooted ‘Merlot’ and ‘Merlot’ vines grafted onto three rootstocks that are commonly used by growers in BC: 3309C, 44-53M, and Riparia Gloire.

## Materials and methods

### Microplot setup

In spring of 2007, 160 field microplots were installed at the Agriculture and Agri-food Canada, Summerland Research and Development Centre (SuRDC) (49°33′45.200″ N; 119°38′55.300″ W). Soil at the site was an Osoyoos loamy sand (Wittneben, 1986). Four 40-m long trenches were dug to a depth of 60 cm, with a between-trench spacing of 3 m. Forty 100 L volume ‘Grip-Lip 10,000’ pots (Nursery Supplies, Inc., Chambersburg, PA) were installed in each trench with a 1 m spacing between the center of each pot. On 17 April 2007, all pots were back-filled with excavated soil to an effective soil volume of 92 L, and the granular fumigant Basamid (active ingredient Dazomet, methyl iso-thionate; Engage Agro Corporation, Guelph, ON, Canada) was incorporated into the soil during backfilling at a rate of 16 g/microplot or 175 g/m^3^ soil. Immediately after backfilling and adding the Basamid, each pot was wetted to saturation and covered with polyethylene film. At four weeks after fumigation (mid-May 2007), the polyethylene film was removed from all microplots and they were allowed to off-gas until nematode inoculation and planting on July 4, 2007. An Environment and Climate Change Canada climate station was located approximately 500 m from the experimental site. Maximum, minimum, and mean daily temperatures for the warmest (July or August) and coldest (December or January) months at the site during the course of the experiment are summarized in Table 1.

**Table 1.** Maximum, minimum, and mean daily temperatures for the warmest (July or August) and coldest (December or January) months at the site during the course of the microplot experiment.

Month, year	Maximum	Minimum	Mean
July, 2007	30.5	16.5	23.5
January, 2008	0.3	−6.6	−3.2
July, 2008	29.2	14.6	21.9
December, 2008	−2.2	−8.5	−5.4
July, 2009	31.1	15.5	23.3
December, 2009	−1.3	−6.5	−3.9
July, 2010	29.5	14.3	21.9
January, 2011	2.0	−4.3	−1.2
August, 2011	29.4	14.1	21.8

### Nematode inoculum

One-half of the microplots, randomly chosen, were each inoculated with 220 *M. xenoplax*. The experimental population used as inoculum was originally isolated from a vineyard block on the grounds of the Summerland Research and Development Centre, approximately 1 km away from the microplot experiment. Subsequent PCR-sequencing of a portion of 28S rDNA confirmed the identity of the population as *M. xenoplax* (NCBI GenBank accession MK176323.1). The population was initiated by inoculating 250 hand-picked *M. xenoplax* onto a rooted cutting of ‘Pinot Grigio’ growing in sterilized sand-based potting medium in a greenhouse pot. The experimental population was subsequently built up over a year via serial transfer onto multiple new self-rooted ‘Pinot Grigio’ plants. For inoculation of the microplots, the potting medium from multiple infested greenhouse plants was combined, mixed, and portioned into 1 L aliquots that were added to the planting hole in each inoculated microplot immediately prior to planting. Non-inoculated control microplots received the same amount of potting medium but from greenhouse-grown ‘Pinot Grigio’ plants that were not infested with *M. xenoplax*. The inoculum was quantified by extracting and counting nematodes from six subsamples of the infested potting medium using the wet-sieving sucrose-centrifugation procedure (Forge and Kimpinski, 2007). Inoculated microplots received an average of 220 *M. xenoplax*. With an average microplot soil volume of 92 L, the initial inoculum translated to an initial population density of 2.4 *M. xenoplax*/L soil.

The plant material was derived from tissue culture and obtained from a commercial nursery where it had been grown in plugs (Bevo Farms, Langley, British Columbia). Twenty of the *M. xenoplax*-inoculated and twenty of the non-inoculated microplots were randomly chosen to be planted with one plant each of the four vine genotypes: (i) self-rooted ‘Merlot’; or ‘Merlot’ grafted onto (ii) 3309C rootstock, (iii) 44-53 rootstock, or (iv) Riparia Gloire rootstock. Thus, the experimental design included 20 replicate microplots of each of the eight combinations of *M. xenoplax* inoculation (+/−) and four rootstocks, arranged in a completely randomized design. Subsequent to planting, trellises and a drip irrigation system were installed in each row, with two drippers positioned on each microplot. Each microplot was fertilized each spring with 10 g N as granular calcium nitrate. Microplots were irrigated as necessary to maintain soil moisture at near field capacity on the basis of daily observations during the growing season.

### Sampling and analyses

A single soil sample was taken from each microplot in late October of 2008, 2009, 2010, and 2011. Each sample was comprised of two 2-cm diameter cores taken to a depth of 30 cm and combined. Nematodes were extracted from a 100 cm^3^ subsample from each sample using wet-sieving sucrose-centrifugation extraction. The *M. xenoplax* in each extract were enumerated using an inverted microscope, and data were expressed as the number of *M. xenoplax*/L soil.

In 2010, the soil samples were passed through a 6 mm sieve prior to nematode extraction to facilitate removal of root fragments. The root fragments were separated into coarse (> 2 mm diameter) and fine (< 2 mm diameter) size classes, dried, weighed, and data expressed relative to dry weight of the soil sample (i.e. g roots/kg dry soil).

In spring of each year, vines were pruned to a single stem that was trained to the top trellis wire at 1.5-m above the ground. The prunings from each vine were collected, dried, and weighed. Vine trunk diameters were measured at 30 cm above the ground in two cardinal directions each winter, and trunk cross-sectional areas (TCSA) were calculated. An unusually early hard freeze occurred in 2009, when temperatures went below −5°C on three successive nights (October, 10, 11, 12). This cold event resulted in the mortality of 44 vines, leaving 116 vines in the experiment through the 2010 and 2011 growing seasons, with 12, 19, 13, and 18 inoculated vines of 44-53, Riparia Gloire, 3309C, and self-rooted vines, respectively, and 11, 16, 12, and 15 non-inoculated vines of 44-53, Riparia Gloire, 3309C and self-rooted vines, respectively. Soil sampling in 2012 revealed *M. xenoplax* contamination of a significant number of control microplots, so the experiment terminated with the plant growth data collected in fall of 2011.

### Statistical analyses

A chi-square test was first used to determine if the fall 2009 to winter 2010 mortality was disproportionately associated with any particular combination of rootstock and *M. xenoplax* inoculation, and no such association was found (*p* = 0.99). Single-factor analysis of variance (ANOVA) was used to assess differences between rootstocks within *M. xenoplax* population densities. Separate analyses were conducted for each year and for the across year averages for each microplot. Plant growth parameters (trunk cross-sectional area, pruning weights, total root biomass, fine root biomass) were subjected to a two-factor ANOVA with nematode inoculation (+/−) and rootstock as the factors. When warranted by significant *M. xenoplax* main factor or rootstock×*M. xenoplax* interaction effects, *t*-tests were used to assess the effect of *M. xenoplax* inoculation for each rootstock separately.

## Results and discussion

### *Mesocriconema xenoplax* population growth

Population densities of *M. xenoplax* increased rapidly on all rootstocks and did not differ significantly among rootstocks in any year (data not shown), or averaged over years (Table 2). These data indicate that all rootstocks were similarly good hosts for *M. xenoplax*. The self-rooted vines tended to support the lowest *M. xenoplax* population densities, however, they also had the smallest fine root biomass (Table 2), suggesting that lower availability of roots rather than inherent resistance was likely the reason for the tendency of self-rooted vines to support smaller *M. xenoplax* populations in this experiment. Overall mean population densities (averaged over rootstocks) increased from 2.4 *M. xenoplax*/L soil at the beginning of the experiment, in July of 2007, to 851, 5,288, 1,226, and 6,451 *M. xenoplax*/L soil in October of 2008, 2009, 2010, and 2011, respectively. This rapid 355X population increase between planting in July of 2007 and October 2008 is comparable to that observed by Pinkerton et al. (2004) on self-rooted ‘Chardonnay’ and ‘Pinot Noir’ in a loamy clay soil in Oregon. In that study, an initial inoculum level of 39 *M. xenoplax*/L soil (assuming a soil bulk density of 1.3 kg/L) increased 205X to approximately 8,000 *M. xenoplax*/L soil by the end of the second growing season. The potential for very small at-plant *M. xenoplax* population densities to increase rapidly and impact vine growth in the second to fourth years after planting suggests that the benefits of pre-plant fumigation would likely be limited to the first one or two years of vine growth, and the concept of threshold population densities to guide management actions may not be practical for *M. xenoplax* on grapevines.

**Table 2.** Effects of rootstock on population densities of *Mesocriconema xenoplax* (Mx) in inoculated microplots and vine growth parameters for ‘Merlot’ grapevines grown in microplots with (+Mx) or without (−Mx) *M. xenoplax.*

	Mx population density	2011 trunk cross-sectional area (mm^2^)		2008-2011 cumulative pruning weights (g dry weight/vine)		Total root biomass (g/kg dry soil)		Fine root biomass (g/kg dry soil)	
Rootstock	Mx/L soil	−Mx	+Mx	−Mx	+Mx	−Mx	+Mx	−Mx	+Mx
44-53	3,559	457	445	135	115	1.47	1.18	1.02	0.91
Riparia Gloire	4,968	340	343	164	153	1.52	1.31	1.21	1.09
3309C	3,985	559*	310	149	153	1.44	1.73	1.11	1.34
Self-rooted	2,172	269**	167	116***	49	1.05*	0.66	0.66***	0.38
Average		406*	316	141***	118	1.37**	1.22	1.00*	0.93

**Notes:** Values for *M. xenoplax* population densities (Mx/L soil) are averages of the 2008, 2009, 2010, and 2011 samples. *Asterisks denote significant differences between +Mx and −Mx microplots for each combination of parameter and rootstock according to *t*-test: *p ≤ 0.05; **p ≤ 0.01; ***p ≤ 0.001.

### Impacts of *M. xenoplax* on vine growth

Rootstock genotype had significant main-factor effects on all vine growth parameters. Because inherent differences between rootstocks in overall growth characteristics has been analyzed and discussed extensively elsewhere (e.g. Keller, 2010), and the focus of this paper is the impacts of *M. xenoplax* on vine growth, this section focuses on main-factor effects of *M. xenoplax* or the *M. xenoplax*×rootstock interaction.

Trunk cross-sectional area (TCSA) is a robust measure of cumulative vine growth and vigor. We found that TCSA was affected by *M. xenoplax* in 2011 but not in preceding years. In 2011, there was a significant *M. xenoplax*×rootstock interaction (*p* = 0.016); inoculation with *M. xenoplax* did not affect TCSA of 44-53 or Riparia Gloire, but reduced TCSA of 3309C by 45% and that of self-rooted vines by 38% (Table 2). Pruning weights were highly variable and there were no significant effects of *M. xenoplax* or the *M. xenoplax* × rootstock interaction on pruning weights in any given year. Analyses of four-year cumulative pruning weights indicated a significant main-factor effect of *M. xenoplax* (*p* = 0.009), and within-rootstock *t*-tests indicated that *M. xenoplax* reduced pruning weights of self-rooted vines in particular (Table 2). These results for self-rooted ‘Merlot’ are similar to results for self-rooted ‘Pinot Grigio’ from a preceding microplot study with the same population of *M. xenoplax* at a nearby site (Smit, 2009). For both fine and total root biomass, there were significant main-factor effects of *M. xenoplax* (*p* = 0.010 and 0.027, respectively), but no *M. xenoplax*×rootstock interaction, and within-rootstock *t*-tests revealed significant effects of *M. xenoplax* inoculation on self-rooted vines but not any of the rootstocks (Table 2).

While all three rootstocks were equally good hosts for this BC population of *M. xenoplax*, the self-rooted vines and 3309C rootstock appeared to be less tolerant to *M. xenoplax* than 44-53M or Riparia Gloire rootstocks. The lack of measurable effects of *M. xenoplax* on 44-53M and Riparia Gloire rootstocks in our study contrasts with Pinkerton et al. (2005), who reported that an initial inoculum density of approximately 1,000 *M. xenoplax*/L soil significantly suppressed growth of both 44-53M and Riparia Gloire over one year in greenhouse pots. Similarly, Schreiner, Pinkerton and Zasada (2012) and Schreiner, Zasada and Pinkerton (2012) demonstrated significant impacts of an initial inoculum density of 1,000 *M. xenoplax*/L soil on growth of 3309C over four years in microplots in Oregon. We suggest that the lack of measurable effects of *M. xenoplax* on 44-53M and Riparia Gloire in our study was due to the lower at-plant *M. xenoplax* population densities. As well, the overwinter vine mortality that reduced the number of vines in the experiment and contributed to vine-to-vine variation likely reduced our statistical power to measure modest effects of *M. xenoplax* on growth of the rootstocks in this study.

Our data are limited to vegetative growth parameters, as contamination of control microplots required termination of the experiment before it became possible to collect reliable fruit yield data. Additional research is needed to provide a more detailed indication of the damage potential of *M. xenoplax,* including impacts on fruit yield and quality, and to identify avenues for its management in BC vineyards. In particular, additional microplot studies with multiple BC populations of *M. xenoplax* and a wider range of the rootstocks, including rootstocks previously identified elsewhere as being at least partially resistant or tolerant to *M. xenoplax* (Pinkerton et al., 2005; Ferris et al., 2012), would yield important practical information for vineyard managers in the region. Rootstocks 420A and 101-14 were found to be resistant and moderately resistant by Pinkerton et al. (2005). In addition, two rootstocks recently released by the University of California-Davis, UCD-GRN1, and UCD-GRN2 appear to also be partially resistant to *M. xenoplax* (Ferris et al., 2012). UCD-GRN1 appears to have the most consistent resistance to *M. xenoplax*, but it is sensitive to temperatures below −5°C (Ferris et al., 2012) and would therefore not be viable for Okanagan Valley vineyards. UCD-GRN5 appears to be partially resistant to some populations of *M. xenoplax* in California but susceptible to others (Ferris et al., 2012); it therefore deserves to be evaluated against BC populations of *M. xenoplax*.

The longer-term impacts of *M. xenoplax* on more mature vines and fruit yields have not been quantified. Additional research linking populations of *M. xenoplax* in mature vineyards to vine vigor and susceptibility to other stresses could greatly enhance overall understanding of factors underpinning sustained productivity of vineyards in the region.

## Conclusions

This study builds on previous studies on fine-textured soils in Oregon (Pinkerton et al., 2004, 2005; Schreiner, Pinkerton and Zasada, 2012; Schreiner, Zasada and Pinkerton, 2012), demonstrating that self-rooted ‘Merlot’ and three commonly used rootstocks are excellent hosts for a BC population of *M. xenoplax,* and that the nematode can in-turn impair early grapevine growth under field conditions typical of the Okanagan Valley. The wine grape industry in BC is relatively young, with a significant portion of acreage planted in the past 30 years on land that was previously either uncultivated or used for other crops. Consequently, plant-parasitic nematodes such as *M. xenoplax* were not previously considered to be significant concerns to the wine grape industry. Due to scarcity of irrigated land in the region that is climatically suitable for wine grape production, future renewal of the industry will increasingly involve replanting into previous vineyard sites, many of which will likely harbor substantial populations of *M. xenoplax*. The potential for rapid *M. xenoplax* population growth suggests that the benefits of pre-plant fumigation would likely be limited to the first one or two years after replanting, and no post-plant nematicides are currently registered for use on grapevines in Canada. Additional research on the longer-term responses of potentially resistant rootstocks to BC nematode populations and growing conditions and the development of alternatives to fumigation for ongoing management of *M. xenoplax* will be needed to foster successful replanting of vineyards in the region.
